# An adapted particle swarm optimization algorithm as a model for exploring premyofibril formation

**DOI:** 10.1063/1.5145010

**Published:** 2020-04-17

**Authors:** William Sherman, Anna Grosberg

**Affiliations:** 1 Center for Complex Biological Systems, University of California Irvine, Irvine, California 92697-2280, USA; 2 Edwards Lifesciences Center for Advanced Cardiovascular Technology, University of California Irvine, Irvine, California 92697-2730, USA; 3 Department of Biomedical Engineering, University of California Irvine, Irvine, California 92697-2715, USA; 4 Department of Chemical and Biomolecular Engineering, University of California Irvine, Irvine, California 92697-2580, USA; 5 NSF-Simons Center for Multiscale Cell Fate Research, University of California Irvine, Irvine, California 92697, USA

## Abstract

While the fundamental steps outlining myofibril formation share a similar scheme for different cell and species types, various granular details involved in the development of a functional contractile muscle are not well understood. Many studies of myofibrillogenesis focus on the protein interactions that are involved in myofibril maturation with the assumption that there is a fully formed premyofibril at the start of the process. However, there is little known regarding how the premyofibril is initially constructed. Fortunately, the protein *α*-actinin, which has been consistently identified throughout the maturation process, is found in premyofibrils as punctate aggregates known as z-bodies. We propose a theoretical model based on the particle swarm optimization algorithm that can explore how these *α*-actinin clusters form into the patterns observed experimentally. Our algorithm can produce different pattern configurations by manipulating specific parameters that can be related to *α*-actinin mobility and binding affinity. These patterns, which vary experimentally according to species and muscle cell type, speak to the versatility of *α*-actinin and demonstrate how its behavior may be altered through interactions with various regulatory, signaling, and metabolic proteins. The results of our simulations invite speculation that premyofibrils can be influenced toward developing different patterns by altering the behavior of individual *α*-actinin molecules, which may be linked to key differences present in different cell types.

## I. INTRODUCTION

Across species, molecular interactions cause myofibrils to transmit forces both within and between neighboring myocytes with extreme precision, allowing for coordinated muscular contractions. This force transmission comes about because of synchronizing interactions across the highly ordered myofibril structure consisting of thick and thin filaments with *α*-actinin forming the mechanical link between thin actin filaments.^1,2^ Myofibrils form via a generalized process whereby each stage coincides with a specific collection of proteins, some of which can be used to track the development from an immature to a mature state. Early stage myofibrils, termed premyofibrils, can be identified by the clusters of *α*-actinin distributed throughout their length.^2,3^ Following their formation, the spacing between these punctate *α*-actinin aggregates, designated as z-bodies, increases leading to *α*-actinin registration and lateral fusion amongst neighboring premyofibrils. This creates the striated patterns found on mature myofibrils referred to as z-lines.^3^ These patterns have been observed in various cell types across multiple species including cardiac cells,^4,5^ skeletal muscle cells,^6,7^ and flight muscle cells.^8,9^ This corresponds to a basic organizational motif common to animals that use their muscles to produce force: the partition of the myofibril into repeating sarcomeres.

Since sarcomeres are the central contractile units of myofibrils in myocytes, much attention has been placed on tracking their formation, beginning with their myofibril precursors.^1,10^ However, despite sharing a similar overall pattern, different morphologies have been observed depending on the species of interest. For instance, there is not only a difference in the initial z-body periodicity but also a difference in the final sarcomere length. In mammalian cardiomyocytes, the distance between *α*-actinin clusters elongates from approximately 1.2 *µ*m for z-bodies to 2 *µ*m for mature z-lines,^2,5,11^ while in *Drosophila* flight muscle cells, the distance increases from around 1.7 to 3.2 *µ*m.^8^ This growth has also been observed in the skeletal muscle of zebrafish though with different initial and final lengths.^12^ However, the way in which the final sarcomere lengths are achieved in each species and muscle type is still unclear.

Sarcomerogenesis studies are typically framed with the assumption that there is a predetermined initial z-body pattern at the start of the maturation process.^2,3^ This is often claimed without reference to how *α*-actinin clusters self-organize into these punctate patterns. In fact, there is disagreement on whether proteins involved in maturation are also involved in the formation of z-bodies.^4,13^ Unfortunately, experiments focusing on early protein coalescence in premyofibrils are scarce resulting in few proposals for exploring the z-body pattern formation. This scarcity is due to the vast number of potential protein interactions that could be involved in early stage myofibrillogenesis, the exploration of which should not be undertaken blindly. Since theoretical models are well-suited for studying phenomena without emphasizing specific interactions, they may be key to addressing this unmet need and helping to guide future experiments.

In this work, we investigated whether *α*-actinin dynamics alone are sufficient to drive the self-organization of clusters into regularly spaced intervals simply through adjustments in individual protein activity. Specifically, we examined if *α*-actinin accumulation can be obtained in developing premyofibrils through an energy minimization mechanism without explicit reference to other proteins. Since the formation of premyofibrils has not been entirely explored, our approach may aid experimentalists in developing a roadmap for prospective studies of early stage myofibrillogenesis. By focusing on the recruitment and interactions between neighboring *α*-actinin clusters in different species and muscle cell types, our model can guide experimentalists toward identifying pattern-inducing factors associated with forming z-bodies. Once specific causal links are identified, exploration of the impact altered premyofibrils have on final sarcomere formation can be undertaken.

## II. RESULTS

Many studies have attempted to decode the complexities associated with the striated patterns found in mature muscle cells.^1,9,14^ Often, emphasis is placed on the proteins involved in the transformation from premyofibrils with identifiable z-bodies into mature myofibrils with distinct z-lines.^2,10,15^ However, this type of exploration does not address what causes *α*-actinin to form a pattern along premyofibrils in the first place. In an effort to gain insight into this phenomenon, we viewed the cell as a mechanical operator which relies on an efficient use of free energy to function including forming the premyofibril architecture. Our phenomenological approach focused on whether pattern formation could be induced without specific reference to other proteins that may be involved. This was investigated by employing a modified particle swarm optimization (PSO) algorithm that utilized the energetic profile of a swarm of *α*-actinin to guide z-body pattern formation.

### A. Convergence of the adapted PSO algorithm

The standard PSO algorithm is a stochastic evolutionary algorithm with the ability to converge to a global optimum even when several local optima exist. To ensure proper convergence, many of the suggestions that have been put forth regarding the convergence of the standard PSO algorithm^16–19^ were integrated into the adapted PSO algorithm in this work. These include the incorporation of a dynamically adjusting inertia weight [Eq. (5)] into the velocity update rule,^16^ setting initial velocities to zero,^18^ and utilizing a swarm population size that is sufficiently large.^19^ The implementation details and equations can be found in the Sec. IV.

**FIG. 1.**
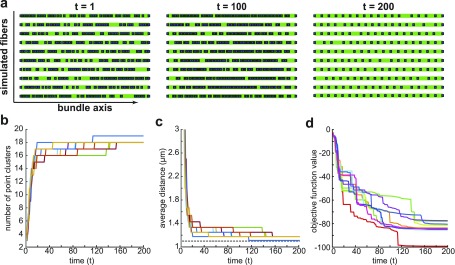
The adapted PSO algorithm converges to the optimal swarm configuration. (a) Ten simulations were run on a horizontal premyofibril (green) with *S* = 60 individual configurations in the swarm. Taken together, the swarm resembled a random distribution of *α*-actinin point clusters (blue). The clusters reached an equilibrium configuration as time increased from *t* = 1 to *t* = 200. (b) As the swarm evolved, new point clusters were incorporated into the swarm throughout all simulations (colors vary) until no new clusters could be added. (c) The inclusion of new clusters caused a decrease in the average inter-cluster distance for each simulation until it converged to a singular value, *r*_*m*_ = 1.1 *µ*m (dotted line). (d) The injection of new point clusters corresponded to a decrease in the objective function value within each simulation.

The initial swarm resembled a collection of *α*-actinin randomly distributed throughout the simulated curve trajectory. Individual configurations were updated according to Eqs. (4)–(8) with the possibility of cluster recruitment whenever the inter-cluster distance of the optimal swarm configuration was sufficiently large. Within each simulation, cluster accumulation can be viewed over the implemented time [Fig. 1(a)]. Random initial swarms each tended toward a final configuration, converging before the maximum iteration counter *T*_max_ was reached [Fig. 1(b)]. The swarm can be observed converging to the optimal swarm configuration with recruitment and movement mechanisms playing a key role in guiding algorithmic convergence. In particular, the final swarm configuration yielded an equilibrium inter-cluster distance near the ideal distance parameter, *r*_*m*_, found in Eq. (2) [Fig. 1(b)]. The reduction in the inter-cluster distance came about as a result of new clusters being recruited to the developing myofibril [Fig. 1(c)]. The incorporation of new clusters into the swarm produced distinct reductions in the objective function value. Despite sharing a similar trend throughout all simulations, the objective function did not decay to a singular steady state value [Fig. 1(d)]. Rather, the energy required to reach an optimal swarm configuration was dependent on the level of randomness and stochasticity in the initial swarm distribution. Many have speculated that cells function in ways which aim to optimize available free energy.^20^ The possibility of an energy state transition occurring as a result of *α*-actinin recruitment invites speculation on the energetic nature of *α*-actinin modulation.

### B. Pattern formation as determined by objective function parameters

A key observation of the convergence tests was the link between *r*_*m*_ and the convergence of the swarming algorithm, suggesting a correlation between the ideal distance value and the effective change in *α*-actinin mobility. Indeed, there are conflicting reports regarding the dynamic movement of *α*-actinin in the early stages of myofibril formation that coincide with the muscle cell type.^4,7^ To explore if it is possible to generate patterns by manipulating *α*-actinin interactions and regulating binding affinity, the parameter pairs governing the objective function were varied. Specifically, focus was placed on the ideal distance parameter *r*_*m*_, which could coincide with the muscle cell type, and the cluster searching distance *d*_*th*_, which defined the necessary spatial distance for cluster recruitment to be considered.

**FIG. 2.**
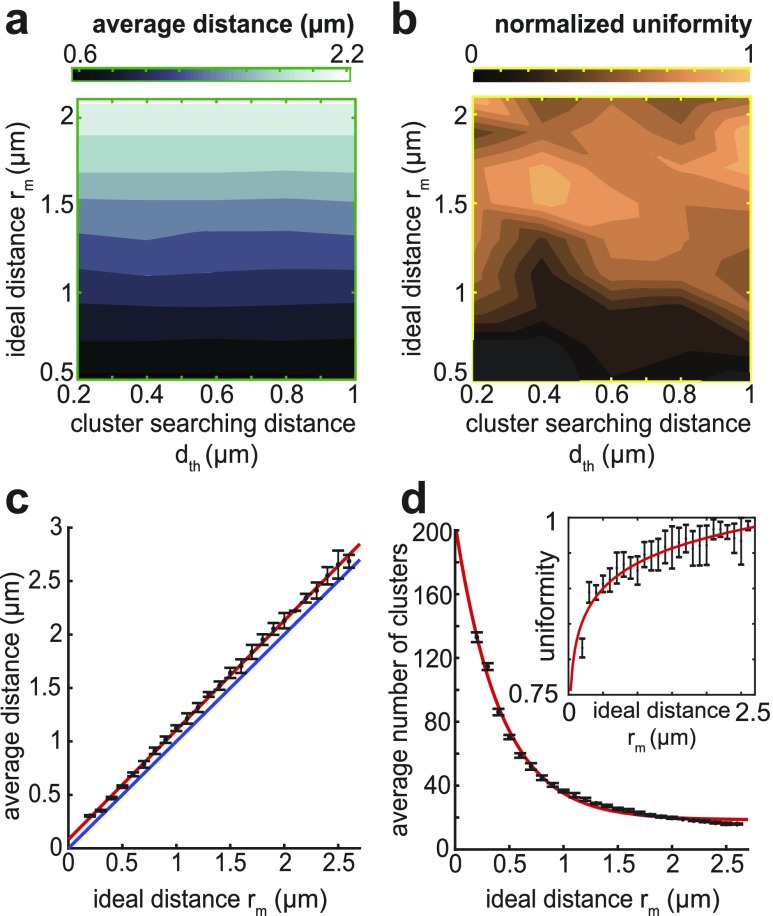
The ideal distance drives convergence behavior. (a) The average inter-cluster distance of the ten fiber simulations showed a correlation between the convergence value and the ideal distance value *r*_*m*_ in the (*d*_*th*_, *r*_*m*_) parameter space. (b) The normalized uniformity measure viewed in the (*d*_*th*_, *r*_*m*_) plane revealed different pattern uniformities based on the location within the parameter space. (c) Fixing *d*_*th*_ = 0.5 *µ*m, the convergence values for each simulation (mean ± SD) behaved linearly with varying *r*_*m*_ (red) with a convergence value always larger than *r*_*m*_ (blue). (d) The increase in the ideal distance corresponded to a non-linear decrease in the number of clusters as well as a non-linear increase in uniformity (inset).

In our simulations, the average inter-cluster distance is shown to be driven primarily by the ideal distance, *r*_*m*_, and not by the recruitment searching distance, *d*_*th*_ [Fig. 2(a)]. This is evident by the final inter-cluster distance converging to values near *r*_*m*_ despite changes to *d*_*th*_. However, alterations in the searching distance impacted the average uniformity of the resulting patterns [Fig. 2(b)]. In particular, the appropriately chosen searching distance could increase uniformity levels for ideal distances larger than approximately *r*_*m*_ = 1.25 *µ*m. For ideal distances below this level, but larger than *r*_*m*_ = 0.7 *µ*m, the searching distance *d*_*th*_ would have little influence, as is evident from the consistent levels of uniformity that were seen. However, pattern formation begins to reduce for ideal distances smaller than *r*_*m*_ = 0.7 *µ*m with the lowest levels occurring for searching distances below *d*_*th*_ = 0.5 *µ*m.

To further explore the influence of the ideal distance term *r*_*m*_, the searching distance was fixed at *d*_*th*_ = 0.5 *µ*m, while *r*_*m*_ was allowed to vary. In this scenario, the final average distance correlated with the changes in the ideal distance in a linear fashion with consistently high levels of uniformity even at low *r*_*m*_ [Fig. 2(c)]. Interestingly, the average distances consistently converged to values slightly larger than the specified ideal distance. Coinciding with the distance convergence, a clear link between the average distance and the number of final point clusters can be observed [Fig. 2(d)]. However, while smaller distances yielded more point clusters, the relationship is non-linear despite a constant searching distance. This appears to inversely mimic the non-linear variation in the uniformity measure [Fig. 2(d), inset], suggesting that uniformity may be more closely linked with the number of clusters than with the final inter-cluster distance. This is a property that has been observed in mature myofibrils where increased uniformity appears to coincide with an uptick in the number of z-lines.^14,21^ Similarly, other properties of mature myofibrils such as sarcomeric length-regulation via specified proteins may have analogs in the immature case.

### C. Impacts of the myofibril shape on pattern initiation

Even though cells exist in a three dimensional (3D) environment, two dimensional (2D) experimental studies are often employed when studying myofibrillogensis,^5,22^ leading to the discovery that premyofibrils first appear near the cell edge.^3^ Despite their potential shape being restricted by the outline of the cell boundary, premyofibrils are often depicted as nearly straight curves with little to no variation in the curvature.^2^ This has inspired many researchers to model components of myofibrillogenesis using one dimensional reductions.^23,24^ One of the advantages of our approach is its adaptability into two dimensional studies without requiring large increases in complexity. This was used to investigate whether the premyofibril shape was a potential influencer of either final inter-cluster distance or pattern uniformity.

**FIG. 3.**
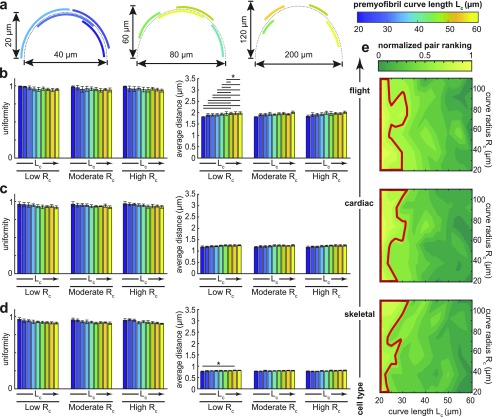
Curve shape influences the pattern development. (a) Fourth-order Bézier curves of various lengths (colorbar) were created for several types of curve radii including low (20 *µ*m), moderate (56 *µ*m), and high (110 *µ*m) values. (b)–(d) For low, moderate, and high curve radii, the curve lengths were varied, and the resulting pattern was quantified according to uniformity (left plots) and the average inter-cluster distance (right plots) as mean ± SD for the ten simulations. Pairings which demonstrated statistically significant differences (*p* < 0.05) were marked with a horizontal line. The simulations were performed using ideal distances corresponding to the muscle cell type: *r*_*m*_ = 1.8 *µ*m for the flight muscle, *r*_*m*_ = 1.1 *µ*m for the cardiac muscle, and *r*_*m*_ = 0.7 for the skeletal muscle. (e) For each muscle cell type, the ranking function (13) was employed and normalized in the *L*_*c*_–*R*_*c*_ parameter space. The region with the ranking value larger than the mean plus one standard deviation for the whole space is outlined (red).

Experiments from the literature emphasize premyofibrils that appear as long, slightly curved rods with distinct punctate patterns. However, the patterns appear to degrade on curves closer to the cell edge, where premyofibrils have a larger radius of curvature.^3,22^ To explore this phenomenon, we applied our algorithm to two dimensional curves of varying lengths and curvatures. The curvature radius *R*_*c*_ was fixed at low (20 *µ*m), moderate (56 *µ*m), or high (110 *µ*m) values, and arc segments were constructed using fourth-order parametric Bézier curves with lengths *L*_*c*_ ranging from 20 *µ*m to 60 *µ*m [Fig. 3(a)]. To perform our study, a linear transformation was applied that aligned the constructed curve with the horizontal axis. This allowed for lateral movement to be determined via Eq. (6) with the corresponding vertical coordinate being determined such that the resulting point cluster remained on the transformed myofibril.

A common trend was seen in all three simulated muscle types (Fig. 3). High levels of uniformity were observed in all cases with no statistical differences found. However, there were differences observed in the final simulation distances at a low curvature radius for the flight and skeletal muscle cell types. In the case of the flight muscle cell, the preassigned length of the myofibril appeared to play a role in the final pattern formation, with longer curves leading to larger deviations from the ideal distance [Fig. 3(b)]. Interestingly, no significant differences were observed in cardiac cells, regardless of the myofibril shape [Fig. 3(c)]. However, the increase can also be seen when the shortest curve length is compared to one of the longer curve lengths in the skeletal muscle cell [Fig. 3(d)]. As in the flight muscle, no statistically significant differences were observed at moderate or high radii of the curvature in the skeletal muscle.

To further examine the link between these two fundamental characteristics, a parameter space exploration was employed [Fig. 3(e)]. For each muscle cell type considered, the myofibril curvature was altered and the resulting length–curvature pair was ranked based on how well the pattern balanced inter-cluster distances and uniformity [Eq. (13)]. In general, increasing the curve length had a detrimental impact on the ranking when the curvature radius was fixed. However, there was no consistency in the nature of this drop off. The highest ranked length–curvature pairs were identified in a cell-specific manner whereby Eq. (13) was employed throughout the parameter space and the region containing pairs with ranking larger than the mean plus one standard deviation were outlined. These high ranking regions differed in all muscle cell types, yet none extended past the ∼33 µm length marker. While flight cells contained two pronounced regions centered at low or moderately high curvatures, cardiac cells contained three protruding regions with a high curvature included in the low and moderately high curvature radii. As the ideal distance reduced, two of these three regions shrank, prioritizing ranking toward straighter curves. Despite these distinct differences, the simulations did not produce any patterns containing an inter-cluster distance outside the ranges reported experimentally. Further exploring the impact of the curve shape on pattern initiation may yield insights into myofibrillogenesis. It is generally accepted that premyofibrils form near the cell periphery, which contain regions of high curvature in spreading cells, and move inward during maturation.^3,22^ Prior to this spatial migration, the lengths and curvatures of the premyofibrils attempting to form in these regions may be subjected to shape constraints that play a vital role in guiding *α*-actinin recruitment and clustering.

### D. Self-organization may be guided by group behavior

As of yet, a unifying mechanism that guides self-organization across multiple species has not been identified. There is speculation, however, that the variability found in the observed patterns may point toward fundamental differences in protein behavior which are species-specific. Indeed, there has been some evidence that *α*-actinin behavior may be altered through interactions with various regulatory, signaling, and metabolic proteins.^25,26^ The methods by which these regulatory interactions are entrenched in some developing cells are not entirely known but they may be linked to key differences present in different cell types.^27^ To explore these potential influencers in our model, cluster behavior profiles were altered by manipulating two acceleration parameters: an individualized cognitive coefficient *c*_1_, which biases behavior toward the best solution for the specific individual, and a group social coefficient *c*_2_ that biases behavior toward the best solution for the swarm. Typically, these values lie in the range 0 ≤ *c*_1_, *c*_2_ ≤ 4 with large values indicating quick movement toward the target goal.^17^ The prescribed behavior profiles influence individual trajectories with *c*_1_ > *c*_2_, indicating a preference toward optimizing individualized self-learning behavior while *c*_1_ < *c*_2_ prioritizes optimization based on group behavior.

**FIG. 4.**
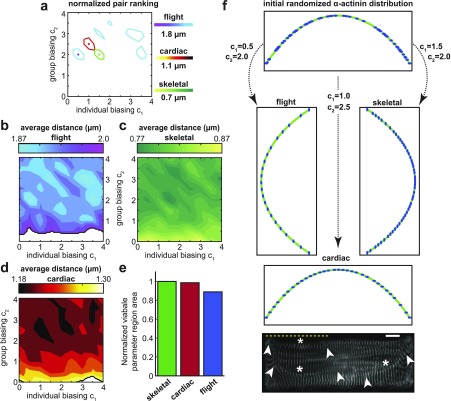
The choice of biasing parameters dictates the equilibrium pattern. (a) The individual (*c*_1_) and group (*c*_2_) biasing behavior parameters were varied for each muscle cell type simulated. The ranking function (13) was normalized, and all regions with values larger than 0.56 were outlined for flight (blue), cardiac (red), and skeletal (green) muscles. (b)–(d) The average inter-cluster distance for each simulated cell type was plotted in the (*c*_1_, *c*_2_) parameter space with blank regions indicating parameter pairs that yielded average inter-cluster distance values outside the range observed experimentally. (e) The area of the parameter space region that yielded biologically relevant patterns was calculated and normalized relative to the area of the entire parameter space for each simulated muscle cell type. (f) From a common initial *α*-actinin distribution, the choice of biasing parameters can produce patterns for each muscle cell type. These are consistent with experimental data such as neonatal rat ventricular cardiomyocytes with z-lines identified by *α*-actinin staining. Curved myofibrils are identified by an asterisk with arrowheads identifying the myofibril trajectory. A yellow dotted line is provided for visual reference of a horizontal line (scale bar = 10 *µ*m).

To examine the influence of biasing behavior on final pattern formation, the pairs of acceleration parameters were varied within the range suggested by the literature.^17^ The simulated myofibril was given a curve length of approximately 35 *µ*m and a radius of curvature of 40 *µ*m, a shape combination which previously fell outside the highest ranked regions for each muscle cell type [Fig. 3(e)]. Using the ranking function, the regions with the highest ranked parameter pairs were identified for each muscle cell type within the parameter phase space [Fig. 4(a)]. The behavior pairings with the largest overall ranking for each cell type were identified and found to be clustered near each other. Often, each behavior type is assumed to have an equal level of influence,^17^ but such pairings were not optimal for any of the cell types simulated. Interestingly, all highly ranked pairs had a group coefficient *c*_2_ above unity but this property was not observed with the self-learning coefficient *c*_1_. As might be expected, there was no region common to all three muscle types but a transitory area could be identified whereby the different muscle types could be simulated with minimal parameter variation [Fig. 4(a)]. Biologically, these observations imply that *α*-actinin may behave differently depending on the muscle cell type. In fact, there is evidence of genetic variability in the different *α*-actinin isoforms in multiple species.^27^ Whether this variability is linked to alterations in *α*-actinin mobility or binding affinity has not been determined.

To further analyze the influence of variations on *α*-actinin behavior, we explored the convergence behavior within the simulations. The regions with the highest rank appeared to correlate with the behavior pairings that produced patterns with a minimal deviation from the idealized distance value [Figs. 4(b)–4(d)]. For the skeletal muscle, all parameter pairs yielded average distance values that were within the range of values previously reported. However, that was not the case for the other two muscle types. In the cardiac simulations, inaccurate patterns were produced when self-learning was highly favored over socialization [Fig. 4(c)], while for the flight muscle, the lack of group interactions (*c*_2_ = 0) prevented the formation of valid patterns [Fig. 4(d)]. This suggests a possible correlation between the idealized distance value *r*_*m*_ and the size of the valid parameter pairings as increases in *r*_*m*_ corresponded to a shrinkage in the potential parameter space [Fig. 4(e)]. This may support the hypothesis that the level of influence different proteins have on *α*-actinin behavior, and the resulting pattern, may vary according to cell or species type.^27^ In any case, in all three cell types considered, it was possible to obtain a final distribution with an average inter-cluster distance near the ideal distance that utilized both self-learning and group biasing (*c*_1_, *c*_2_ ≥ 1). However, doing so could result in distributions with impaired uniformity. While some high ranking regions could be found for each cell type when individual behavior was prioritized (*c*_1_ > *c*_2_), more high ranking regions were produced when emphasis was placed on group behavior (*c*_2_ > *c*_1_), suggesting that the ability of *α*-actinin to interact with neighboring proteins is essential for pattern formation.

Figure 4(a) illustrates how *α*-actinin dynamics can be modulated in order to form various punctate configurations. The experimentally observed patterns could be recreated through the appropriate choice of biasing parameters. Specifically, within each high ranking region, the parameter pairs that yielded the highest ranking values were chosen to demonstrate how the premyofibril assembly may differ in different cell types [Fig. 4(f)]. Starting with an initially random distribution, each pair of biasing parameters was implemented, and the final *α*-actinin distributions were obtained. These distributions produced patterns which had high levels of uniformity and average inter-cluster distances that resembled the values found in the literature. Movement toward different parameter space regimes may come about by modulating protein interactions. This may be done in a species- or cell-specific manner, prompting further analysis of the interactions that allow each cell type to uniquely regulate protein behavior.

## III. DISCUSSION

There are many aspects regarding the dynamics governing myofibrillogensis that remain unresolved. In most studies, emphasis is placed on the proteins affiliated with the transition from z-bodies to z-lines following the identification of fully formed premyofibrils. However, there is currently no consensus on how such a structure is formed. To aid experimentalists in this endeavor, we constructed a modified PSO algorithm and demonstrated that it may be possible to generate experimentally observed patterns through manipulation of the underlying mobility, recruitment, and binding dynamics of *α*-actinin. By utilizing energy-state transitions and allowing for premyofibrils of various lengths and radii of curvature, we were able to explore how relations such as curve shape and biasing behavior may influence the formation of *α*-actinin patterns.

Since the sarcomere is the central contractile unit in a myofibril, many theoretical and experimental models concerned with the sarcomeric organization are built on the view that self-organization comes about due to tension-mediated interactions between actin and myosin filaments.^23,28,29^ While these models accurately display tension as an important factor in guiding maturation, they often ignore the central role *α*-actinin plays in z-body formation. Our approach differs by emphasizing *α*-actinin dynamics, which has been shown to display differences in mobility depending on the muscle cell type.^4,7^ While other models have not attempted to recreate this property, we were able to mimic this response by considering different behavior profiles. Additionally, previous models favored one dimensional simplifications,^23,24^ whereas our approach allows for two dimensional studies with minimal additions. Experimental data obtained from 2D cultures are commonly used and have demonstrated complex cytoskeletal networks consisting of myofibrils with varying curvatures.^3,24^ Previous models have not been set up to address how this factor influences premyofibril formation, while our model allows for the inclusion of curve shape considerations. Since muscle cells *in vivo* are cylindrical in shape,^30^ it is possible that the 3D structure of developing myofibrils may influence the pattern formation.^31^ Several studies have attempted to decode the complex 3D structure that appears in the later stages of myofibril maturation, referred to as the z-disk.^2,5,32^ However, given the increase in experimental complexity associated with developing 3D cultures,^31,33^ two dimensional computational studies such as ours provide a basis for hypothesis testing with the possibility for extensions in the future. Future extensions of our approach would include three dimensional studies whereby the given premyofibril can have a planar curvature as the result of rotations into the *z*-axis. This additional degree of spatial freedom may be included directly, allowing for three dimensional visualization, or indirectly where a three dimensional curve can be projected onto a two dimensional plane.

Our model proposes that premyofibrils can be influenced toward developing different punctate patterns by altering the behavior of individual *α*-actinin molecules. In the same way that a list of maturation-affiliated proteins has been assembled,^2^ our results highlight how a catalog of *α*-actinin influencers in developing premyofibrils also needs to be compiled. One such influencer that has already been discovered in eukaryotic cells is cofilin. This protein has been shown to increase the cross-linking of actin filaments by increasing the number of potential *α*-actinin binding sites.^25^ Such an influence would correspond to a change in the biasing parameters in our model (represented by *c*_1_ and *c*_2_). It is possible that the dynamics governing this increase in binding sites are linked to the changes in mobility in different muscle cell types, but this has yet to be examined.

Based on our framework, our model suggests that experiments focusing on the sequence of events leading to premyofibril formation prioritize the nature of initial *α*-actinin recruitment and its relation to the scaffolding proteins that bind to actin filaments. There is already evidence that proteins such as N-RAP interact with actin filaments prior to the recruitment of *α*-actinin but do not appear to drive sarcomere formation.^4,13^ These types of proteins may be linked to our cluster searching distance *d*_*th*_, influencing *α*-actinin recruitment dynamics and eventual pattern formation. Additionally, the current model can be used as a building block toward linking the formation of *α*-actinin z-bodies with the known interactions that occur during maturation. Experimentalists can use our phenomenological findings to inform their explorations of the dynamics that are at play during the early stages of myofibrillogenesis. By emphasizing the dynamics of *α*-actinin, our model can be extended to include additional cell types not considered here such as vertebrate smooth muscle. Smooth muscle cells contain *α*-actinin z-bodies but have a different contractile mechanism than striated muscle cells.^30^ This exemplifies how exploring initial *α*-actinin pattern formation phenomenologically may be advantageous from a modeling perspective as it allows for a discussion of general characteristics that may be applicable to many different cell types.

## IV. METHODS

**FIG. 5.**
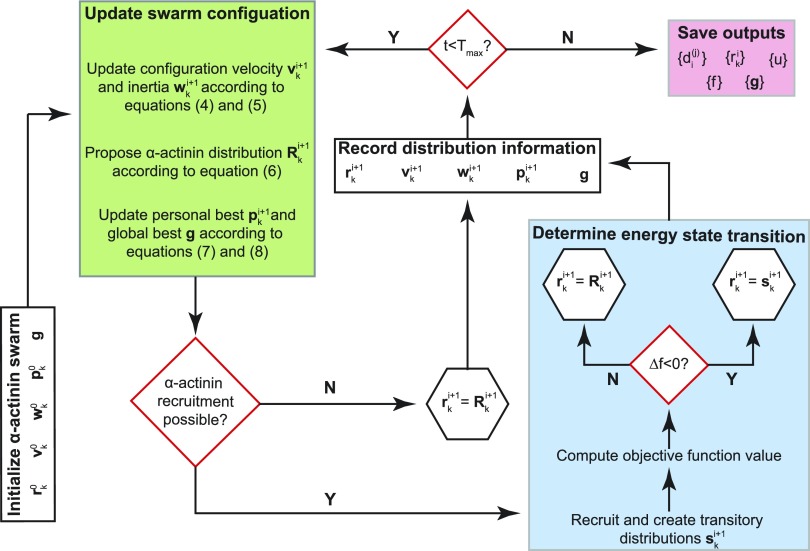
Overview of the adapted PSO algorithm. Following initialization, the algorithm can be broken down into two main components: the position- and velocity-related update equations (green box, described in Sec. IV C 2) and the integrated energy state transition process (blue box, described in Sec. IV C 3).

The PSO algorithm is a population-based algorithm inspired by the social behavior observed in bird flocks and fish colonies.^17^ A key component of this algorithm is its ability to use local interactions between neighboring bodies to influence global behavior in the service of optimizing a prescribed objective function. In our formulation, a swarm refers to a collection of *α*-actinin cluster configurations, each of which may form as the result of an accumulation of *α*-actinin proteins along the simulated myofibril. The *α*-actinin distribution for each configuration was then updated iteratively by optimizing the objective function, as outlined in Fig. 5.

### A. Energy-based objective function

We constructed an objective function that utilized the inter-cluster distance within a configuration and accounted for the energetic cost-benefit of adding clusters to each configuration. Newly added clusters allowed the swarm to consider different configurations where a new state was adopted if it was energetically favorable. Assuming a collection of *N* clusters with locations **r**_1_, …, **r**_*N*_ within a given configuration, we wrote the objective function at iteration *t* as

$$f\left(r_{1},\;\ldots ,\;r_{N}\right)=\underset{i<j}{\sum }V\left(r_{ij}\right)+\overset{K_{l}}{\underset{k=1}{\sum }}E_{k}\delta_{k},$$ (1)

where the first sum denoted the energetic cost of maintaining the swarm in the current state and the second sum denoted the energetic cost-benefit of adding new *α*-actinin clusters to the current swarm.

The potential energy function *V* was chosen under the assumption that *α*-actinin clusters aim at achieving an optimal distance from neighboring clusters, as has been reported experimentally.^2,11^ This behavior is adequately captured by the Lennard–Jones potential energy function,

$$V\left(r_{ij}\right)=\varepsilon \left[{\left(\frac{r_{m}}{r_{ij}}\right)}^{12}-2{\left(\frac{r_{m}}{r_{ij}}\right)}^{6}\right],$$ (2)

where the strength of the cluster interactions is dependent on the inter-cluster distance *r*_*ij*_ = |**r**_*i*_ − **r**_*j*_|, *ε* denotes the depth of the potential energy well, and *r*_*m*_ denotes the ideal equilibrium inter-cluster distance. In the second summation, *K*_*l*_ denotes the number of potential clusters that could be added to the swarm with *E*_*k*_ denoting the energetic cost-benefit of incorporating a new cluster **s**_*k*_ to the swarm configuration. The step function *δ*_*k*_ indicates whether adding **s**_*k*_ is energetically favorable.

### B. Energetic cost-benefit function

To incorporate a new *α*-actinin cluster **s**_*k*_ into a configuration, both the current optimal swarm configuration {**r**_1_, …, **r**_*N*_} and the newly incorporated clusters {**s**_1_, …, **s**_*m*_}, where *m* < *k*, were considered. To this end, we wrote $X=\left\{\left({\tilde{r}}_{0},{\tilde{r}}_{1}\right),\left({\tilde{r}}_{1},{\tilde{r}}_{2}\right),\;\ldots ,\;\left({\tilde{r}}_{N},{\tilde{r}}_{N+1}\right)\right\}$, where **r**_0_ and **r**_*N*+1_ denoted the two endpoints of the myofibril and ${\tilde{r}}_{i}\in \left\{Tr_{0},\;\ldots ,\;Tr_{N+1}\right\}$ with ${\tilde{r}}_{i,x}<{\tilde{r}}_{i+1,x}$. The linear transformation *T* aligned the myofibril of interest with the *x*-axis so that a pseudo-ordering of the clusters could be created. We then defined *E*_*k*_ as

$$\begin{array}{ccc} E_{k}=\underset{\left(x_{1},x_{2}\right)\in X}{\sum }P\left(s_{k},x_{1},x_{2}\right)\left[\overset{N}{\underset{j=1}{\sum }}V\left(\left|s_{k}-r_{j}\right|\right)+\underset{m<k}{\sum }V\left(\left|s_{k}-s_{m}\right|\right)\delta_{m}\right], & & \end{array}$$ (3)

with

$$P\left(s_{k},x_{1},x_{2}\right)=\left\{\begin{array}{cc} 1,\; & {\left(Ts_{k}\right)}_{x}\in \left(x_{1,x},x_{2,x}\right)\\ \; & \text{ and}{\left(Ts_{m}\right)}_{x}\notin \left(x_{1,x},x_{2,x}\right)\text{for}m<k\\ 0,\; & \text{otherwise} \end{array}\right..$$

The first summation within the brackets considers how a newly proposed cluster would influence the current optimal configuration, while the second summation incorporates the influence of clusters which have already been proposed. The outer summation and the function *P* restrict only one cluster to be added to a myofibril segment per iteration.

### C. Algorithm overview

Full implementation was achieved by using a three step process. Following initialization, configurations within the swarm were updated using position and velocity equations common to standard PSO algorithms, as described below (Fig. 5, green box). Once new configurations were proposed, the simulated myofibril was segmented into potential *α*-actinin recruitment zones whereby an energy state transition could occur (Fig. 5, blue box). This process was repeated until the maximum number of iterations *T*_max_ was reached. The final configurations were then analyzed for pattern formation and consistency (Fig. 5, pink box).

#### 1. Initialization

The swarm was initialized by specifying a swarm size *S* and assuming there was initially one cluster on either end of the myofibril curve and one cluster placed randomly within the curve per configuration. The algorithmic variables were initialized by assuming each cluster initially had zero velocity and was in its locally optimal configuration: **v**_*k*_ = 0 and **p**_*k*_ = **r**_*k*_ for all *k* = 1, …, *S*. The optimal configuration for the swarm was initially set to be the most energetically favorable of all the individual configurations: **g** = **r**_*k*_ such that *f*(**r**_*k*_) ≤ *f*(**r**_*j*_) for all *j* = 1, …, *S*.

#### 2. Configuration updates

While *t* < *T*_max_, all algorithmic variables were updated according to the following rules:

1. For *k* = 1, …, *S*, **v**_*k*_ was updated using

$$v_{k}\left(t+\Delta t\right)=\omega \left(t\right)v_{k}\left(t\right)\;+\;R_{1}c_{1}\left(p_{k}-r_{k}\left(t\right)\right)\;+\;R_{2}c_{2}\left(g-r_{k}\left(t\right)\right).$$ (4)

The parameters **R**_1_ and **R**_2_ were random values chosen from a uniform distribution, while *c*_1_ and *c*_2_ denoted scalar weights that bias the attraction toward **p**_*k*_ and **g**. The inertial parameter *ω* is dynamically adjusted at each iteration via the relation

$$\omega \left(t\right)=\omega_{\max}-\left(\omega_{\max}-\omega_{\min}\right)\frac{t}{T_{\max}},$$ (5)

where *ω*_min_ was the final value of *ω* and *ω*_max_ was the initial value of *ω*, typically taken near 1.^17^

2. For *k* = 1, …, *S*, **r**_*k*_ was updated using

$$r_{k}\left(t+\Delta t\right)=r_{k}\left(t\right)+v_{k}\left(t+\Delta t\right)\Delta t.$$ (6)

3. For *k* = 1, …, *S*, **p**_*k*_ and **g** were updated using

$$p_{k}\left(t+\Delta t\right)=\left\{\begin{array}{cc} p_{k}\left(t\right),\; & f\left(r_{k}\left(t+\Delta t\right)\right)\geq f\left(p_{k}\left(t\right)\right)\\ r_{k}\left(t+\Delta t\right),\; & f\left(r_{k}\left(t+\Delta t\right)\right)<f\left(p_{k}\left(t\right)\right) \end{array}\right.$$ (7)

and

$$g=r_{k}\left(t+\Delta t\right)$$ (8)

such that *f*(**r**_*k*_(*t* + Δ*t*)) ≤ *f*(**r**_*j*_(*t* + Δ*t*)) for all *j* = 1, …, *S*.

#### 3. Energy state transition

After new configurations were obtained, we determined if the swarm should undergo a state transition by adding new clusters. If the distance between neighboring clusters in the optimal swarm configuration was larger than the minimum required segment length *d*_*th*_, then new clusters could be incorporated randomly onto the myofibril segment between them. A newly suggested cluster **s**_*k*_ was accepted into the swarm if its inclusion would result in a reduction in the objective function value, Δ*f* < 0. Once a new cluster point within a myofibril segment was accepted into the optimal swarm configuration, a corresponding local cluster **r** was randomly placed within the same myofibril segment for each individual configuration. Each of these new local clusters was initialized using the same requirements as before: **p** = **r** and **v** = 0.

Within each simulation, we allowed for movement in one dimension of each cluster to be determined by the algorithm and required the corresponding second dimension to be chosen such that the cluster remained on the given myofibril curve. We also required **r**_*k*_ to always be confined within the designated boundaries by enforcing absorbing boundary conditions: If **r**_*k*_ was predicted to move a cluster beyond the boundary, then the cluster was reset to the boundary and its velocity was reset to 0.

### D. Distance, uniformity, and ranking measurements

#### 1. Average inter-cluster distance

For pseudo-ordered cluster points {**x**_1_, …, **x**_*N*_} in the *j*th configuration (1 ≤ *j* ≤ *S*), the inter-cluster distance between clusters **x**_*i*_ and **x**_*i*+1_ was defined as $d_{i}^{\left(j\right)}=|x_{i}-x_{i+1}|$. The average distance within the simulation number *s*_*k*_ was ${\bar{d}}_{s_{k}}=\left(1/S\right)\sum_{j}{\bar{d_{i}}}^{\left(j\right)}$, where ${\bar{d_{i}}}^{\left(j\right)}=\left(1/N\right)\sum_{i}d_{i}^{\left(j\right)}$ was the average distance of the clusters located at {**x**_1_, …, **x**_*N*_}. Given *N*_sim_ simulations, the average inter-cluster distance of the swarming algorithm was

$$\bar{d}=\frac{1}{N_{\text{sim}}}\underset{s_{k}}{\sum }{\bar{d}}_{s_{k}}.$$ (9)

To aid in parameter space exploration whereby a pair of parameters (*p*_1_, *p*_2_) was varied, the average inter-cluster distance of the swarming algorithm was rescaled relative to the ideal distance *r*_*m*_,

$${\bar{d}}_{T}\left(p_{1},p_{2}\right)=\frac{\bar{d}\left(p_{1},p_{2}\right)-r_{m}}{\underset{\left(q_{1},q_{2}\right)}{\max}|\bar{d}\left(q_{1},q_{2}\right)-r_{m}|}.$$ (10)

#### 2. Uniformity

To determine whether the point clusters were uniformly distributed, an adjusted coefficient of variation (COV) measure was utilized. To this end, the minimum distance between point **x**_*i*_ and all other points was first determined: *γ*_*i*_ = min_*j*≠*i*_|**x**_*i*_ − **x**_*j*_|. The uniformity measure was then defined as

$$u=\frac{1}{\lambda_{\text{rand}}}\left[\lambda_{\text{rand}}-\lambda \right],$$ (11)

where $\bar{\gamma }=\left(1/N\right)\sum_{i}\gamma_{i}$ and $\lambda =\left(1/\bar{\gamma }\right){\left[\left(1/N\right)\sum_{i}{\left(\gamma_{i}-\bar{\gamma }\right)}^{2}\right]}^{1/2}$, a common COV measure of uniformity. The parameter *λ*_rand_ denotes the maximum uniformity measure that may occur from 60 simulations of *N* randomly distributed points along the simulated myofibril. By including this parameter, the uniformity measure was expected to range from zero to unity with *u* = 1 corresponding to a uniformly distributed collection of points. As with the inter-cluster distance measurements, for each simulation *s*_*k*_, the average uniformity was denoted ${\bar{u}}_{s_{k}}$ and the average uniformity of the swarming algorithm was defined as the average uniformity over all simulations,

$$\bar{u}=\frac{1}{N_{\text{sim}}}\underset{s_{k}}{\sum }{\bar{u}}_{s_{k}}.$$ (12)

#### 3. Ranking function

To determine the influence of a pair of parameters (*p*_1_, *p*_2_) on the behavior of the resulting configuration, a ranking function was constructed which utilized the average inter-cluster distance and uniformity measurements. For each parameter pairing, the distance measurement (10) was normalized and rescaled to prioritize values closer to zero and penalize values away from zero, regardless of whether they deviated toward the positive or negative ends of the spectrum. Thus, the ranking function was defined as the product of the average uniformity and the prioritized distance function,

$$R\left(p_{1},p_{2}\right)=\bar{u}\left(p_{1},p_{2}\right)\;\cdot \;\exp\left[-\frac{\bar{d}{\left(p_{1},p_{2}\right)}^{2}}{2\sigma^{2}}\right],$$ (13)

where *σ* is the standard deviation of the collection of parameter-generated distances ${\left\{{\bar{d}}_{T}\right\}}_{\left(p_{1},p_{2}\right)}$.

### E. Statistical analysis

Simulation data, when applicable, were expressed using the mean with error bars representing the standard deviation. Statistical significance between data groups was determined using one-way analysis of the variance followed by the Tukey–Kramer post-hoc test for pairwise comparisons. A *p*-value less than 0.05 was considered statistically significant.
